# Supplementary material for: Splice-Junction-Based Mapping of Alternative Isoforms in the Human Proteome
Source: Cell Rep. Author manuscript; Available in PMC 2020 Jan 15. (PMC6961840; doi:10.1016/j.celrep.2019.11.026)
Supplement: 2 [file NIHMS1546469-supplement-2.zip › DF1/PXD010154/AdrenalGland/AdrenalGland_19_ZHX3_SWSLSSQPLPSLLTLPCMIPVK.pdf]

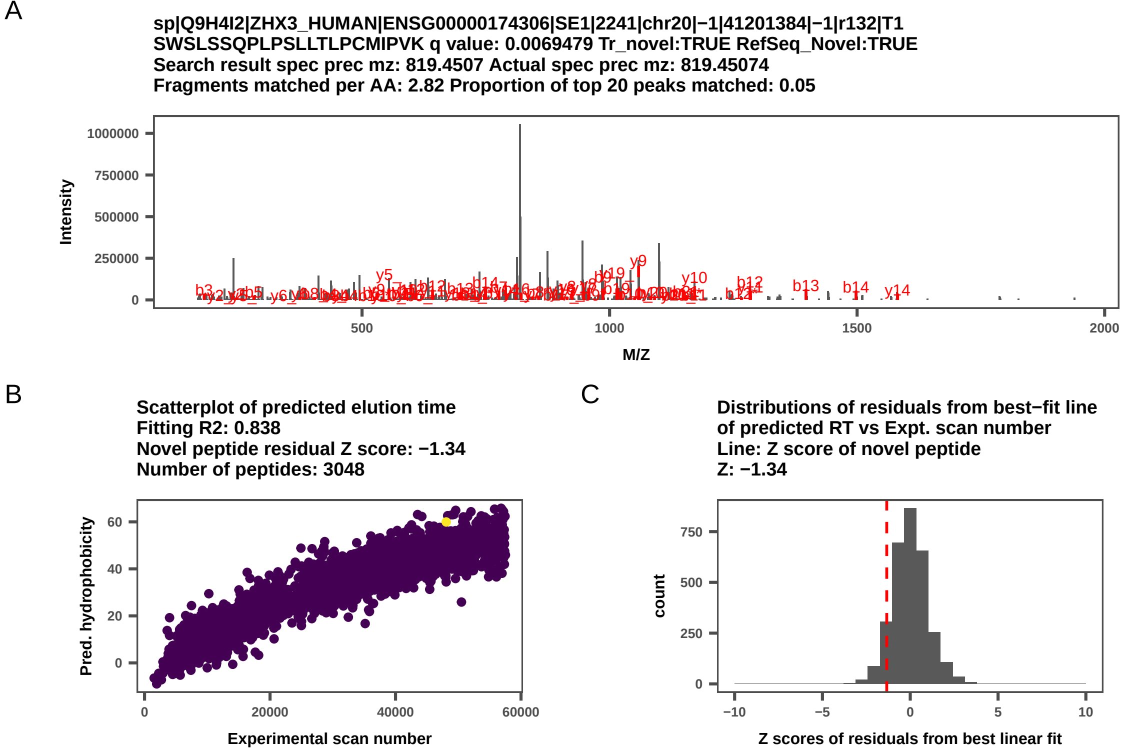
A
sp|Q9H4I2|ZHX3_HUMAN|ENSG00000174306|SE1|2241|chr20|−1|41201384|−1|r132|T1
SWSLSSQPLPSLLTLPCMIPVK q value: 0.0069479 Tr_novel:TRUE RefSeq_Novel:TRUE
Search result spec prec mz: 819.4507 Actual spec prec mz: 819.45074
Fragments matched per AA: 2.82 Proportion of top 20 peaks matched: 0.05
Intensity
1000000
750000
500000
250000
0
M/Z
500
1000
1500
2000
B
Scatterplot of predicted elution time
Fitting R2: 0.838
Novel peptide residual Z score: −1.34
Number of peptides: 3048
Pred. hydrophobicity
60
40
20
0
Experimental scan number
0
20000
40000
60000
C
Distributions of residuals from best−fit line
of predicted RT vs Expt. scan number
Line: Z score of novel peptide
Z: −1.34
count
750
500
250
0
Z scores of residuals from best linear fit
−10
−5
0
5
10
